# Endogenous Levels of Alpha-Synuclein Modulate Seeding and Aggregation in Cultured Cells

**DOI:** 10.1007/s12035-021-02713-2

**Published:** 2022-01-04

**Authors:** Eftychia Vasili, Antonio Dominguez-Meijide, Manuel Flores-León, Mohammed Al-Azzani, Angeliki Kanellidi, Ronald Melki, Leonidas Stefanis, Tiago Fleming Outeiro

**Affiliations:** 1 Department of Experimental Neurodegeneration, Center for Biostructural Imaging of Neurodegeneration, University Medical Center Goettingen, 37073 Goettingen, Germany; 2 Laboratory of Neuroanatomy and Experimental Neurology, Department. of Morphological Sciences, CIMUS, IDIS, University of Santiago de Compostela, Santiago de Compostela, Spain; 3 Departamento de Medicina Genómica Y Toxicología Ambiental, Instituto de Investigaciones Biomédicas, Universidad Nacional Autónoma de México, AP 70-228, 04510 México, DF Mexico; 4 Institut Francois Jacob (MIRCen), CEA, and Laboratory of Neurodegenerative Diseases, CNRS, Fontenay-Aux-Roses, France; 5 Biomedical Research Foundation of the Academy of Athens, 11527 Athens, Greece; 6 Max Planck Institute for Experimental Medicine, Goettingen, Germany; 7 Translational and Clinical Research Institute, Faculty of Medical Sciences, Newcastle University, Framlington Place, Newcastle Upon Tyne, NE2 4HH UK; 8 Scientific Employee With a Honorary Contract at Deutsches Zentrum Für Neurodegenerative Erkrankungen (DZNE), Göttingen, Germany

**Keywords:** PD, LBs, aSyn, Aggregation, Phosphorylation

## Abstract

Parkinson’s disease is a progressive neurodegenerative disorder characterized by the accumulation of misfolded alpha-synuclein in intraneuronal inclusions known as Lewy bodies and Lewy neurites. Multiple studies strongly implicate the levels of alpha-synuclein as a major risk factor for the onset and progression of Parkinson’s disease. Alpha-synuclein pathology spreads progressively throughout interconnected brain regions but the precise molecular mechanisms underlying the seeding of alpha-synuclein aggregation are still unclear. Here, using stable cell lines expressing alpha-synuclein, we examined the correlation between endogenous alpha-synuclein levels and the seeding propensity by exogenous alpha-synuclein preformed fibrils. We applied biochemical approaches and imaging methods in stable cell lines expressing alpha-synuclein and in primary neurons to determine the impact of alpha-synuclein levels on seeding and aggregation. Our results indicate that the levels of alpha-synuclein define the pattern and severity of aggregation and the extent of p-alpha-synuclein deposition, likely explaining the selective vulnerability of different cell types in synucleinopathies. The elucidation of the cellular processes involved in the pathological aggregation of alpha-synuclein will enable the identification of novel targets and the development of therapeutic strategies for Parkinson’s disease and other synucleinopathies.

**Supplementary Information:**

The online version contains supplementary material available at 10.1007/s12035-021-02713-2.

## Introduction

Misfolding and aggregation of aSyn into large intraneuronal inclusions called Lewy bodies (LBs) and Lewy neurites (LNs) is a common feature and a defining hallmark of Parkinson’s disease (PD) and other related neurodegenerative diseases known as synucleinopathies [1]. Neuropathologically, aSyn pathology seems to progress throughout interconnected brain regions in a pattern consistent with the “prion hypothesis” for the spreading of protein pathology [2]. Cell-to-cell transmission of aSyn pathology involves the conversion of the endogenous protein into a pathological conformation, thus propagating the pathology. However, the exact mechanisms involved remain unclear. Mounting evidence suggests that the intrinsically disordered monomeric aSyn assembles into small soluble oligomers, with increased *β*-sheet content that are prone to further aggregation and that may, along the way, result in the formation of toxic species [3, 4].

Several in vivo studies described the injection of aSyn-containing brain tissue homogenates from patients or from transgenic mice, or of in vitro assembled preformed fibrils (PFFs) of recombinant aSyn into mouse brains, blood, or into other organs. The injected animals develop aggregates containing endogenous aSyn phosphorylated on Ser129 in brain regions directly or indirectly connected with the injection site, time-dependent neuronal loss, neuroinflammation, and behavioral alterations [5].

In addition, small seeds of PFFs are internalized in primary neuronal cultures and promote aggregation of endogenous aSyn phosphorylated on Ser129, alongside with progressive impairment in neuronal network function that eventually leads to neuronal death [6, 7]. These PFF-based models have been extensively used to investigate the mechanisms by which aSyn seeds are transferred from donor to recipient cells, thereby spreading throughout the brain. However, the precise molecular mechanisms involved in the uptake, seeding, and aggregation of aSyn are still elusive.

In our study, we tested the hypothesis that the susceptibility to the accumulation of pathological aSyn might be influenced by the endogenous levels of expression of the protein. This arises from the fact that, in the human brain, different cell populations appear to be particularly sensitive in PD. To test our hypothesis, we utilized stable cell lines expressing wild type (WT) aSyn, incubated with a low concentration of PFFs in order to recapitulate aSyn seeding. The development of our model aimed at the comparative study among cell lines with different endogenous levels of aSyn in order to investigate the impact on aSyn uptake and formation of endogenously aggregated aSyn species.

## Materials and Methods

### Recombinant Protein Preparation

For the production of recombinant aSyn protein from *E.coli*, we followed established procedures [8, 9]. For the purification of recombinant aSyn, the protein was first filtered through a 0.22-µm syringe filter and then applied to Hi Trap Q HP anion exchange column (GE Healthcare Life Sciences) equilibrated with 25 mm Tris, and pH 7.6. aSyn was eluted by running a linear gradient of elution buffer (3 M NaCl in 25 mm Tris, pH 7.6) up to 30% elution buffer. aSyn-enriched fractions (analyzed by SDS-PAGE and Coomassie staining/destaining) were collected, combined, and concentrated with 3-kDa molecular weight cut-off Amicon Ultra Centrifuge filters according to Manufacturer’s instructions. Proteins were further purified by Superdex 75 column (GE Healthcare Life Sciences) equilibrated with 25 mM Tris, pH 7.6, and 100 mm NaCl. Pure fractions corresponding to monomeric state (as determined by Native Gel, Coomassie staining/destaining) were combined, concentrated at a final concentration of 5 mg/ml, and stored at − 80 °C until further use. PFFs were prepared from the monomers according to standard protocols [6].

### Generation of the Inducible Stable Cell Line for aSyn Oligomerization Based on Bimolecular Fluorescence Complementation (BiFC)

Naïve HEK293 cells were first transfected with the pTet-Off (Clontech) plasmid that expresses the regulatory protein tTA (tetracycline-controlled transactivator, tTA), which contain a neomycin-resistance gene for the selection. Selection with G418 (500 μg/ml; Millipore) was used for the selection of resistant colonies. Inducibility of resistant colonies was determined after transient transfection with the firefly luciferase vector pTRE-Tight-Luc, in presence or absence of Doxycycline (Dox, 2 μg/ml; Sigma-Aldrich). A clonal HEK293-pTet-Off cell line was selected and used for the generation of the different pTRE stable cell lines (pTRE-Tight-VN-aSyn and pTRE-Tight-aSyn-VC). Transient co-transfection for the pTRE-Tight-VN-aSyn and pTRE-Tight-aSyn-VC vectors was used to verify the formation of the BiFC oligomers. The VN and VC aSyn constructs were generated from initial plasmids containing the VN and VC fused aSyn proteins, digested with the SnaBI/XbaI enzymes and cloned into the pTRE-Tight vector (Clontech) digested before with SmaI/XbaI. The positive clones were verified by DNA sequencing. Subsequently, the VN and VC fused aSyn vectors were co-transfected with a linear selection marker for Hygromycin (Clontech). Selection was performed with 50 μg/ml Hygromycin (Roche). Resistant clones were picked and aSyn was examined by immunoblotting in the presence or absence of Dox (2 μg/mL added for more than 5 days) using a specific antibody against aSyn (anti-aSyn Syn-1 mouse, BD Transduction Laboratories).

### Generation of HEK293-aSynEGFP Stable Cell Line

HEK293 were transfected with a plasmid encoding human WT aSyn fused to EGFP, at the C-terminus, driven by the cytomegalovirus (CMV) promoter. The plasmid contained a selection marker for the antibiotic geneticin (G418) which was used for the selection of the stable transformants. Protein expression was confirmed by Western blot analysis and fluorescence microscopy. A clonal HEK293-aSynEGFP cell line was selected and used for subsequent experiments.

### Construction of Lentivirus Vectors

Full-length human aSyn was PCR-amplified and sub cloned into a 3rd-generation lentiviral plasmid with hUbC-driven EGFP, where the original GFP cassette has been replaced by the aSyn gene including the Kozak sequence. pFUGW vector containing only the GFP cassette was used for control experiments (Addgene) [10].

### Cell Culture and Cell Treatment

HEK293 cells were maintained in DMEM media supplemented with 10% Fetal bovine serum gold (FBS) (PAA) and 1% penicillin–streptomycin (PAN). The cells were grown at 37 °C in an atmosphere of 5% CO_2_. For the seeding experiments, aSyn monomers and PFFs were diluted in PBS, fragmented by sonication in case of PFFs [6], and then added to the cells at final concentration of 100 nM. Control cells were exposed to vehicle only (PBS). Cells were further incubated for 48 h.

### Primary Cultures of Cortical Neurons

Primary cortical neurons were prepared from embryonic day E16-E17 mouse brains. Briefly, after dissection, the tissue was washed three times with Hank’s Balanced Salt Solution (HBSS; Gibco) and digested by Trypsin (Gibco) for 15 min at 37 °C, followed by addition of 100 μl DNase I (10 mg/ml; Roche) and 100 μl FBS (Life Technologies), mixed by inverting and centrifuge at 500 rpm for 5 min. After centrifuge trypsin solution was removed and replaced with 1 ml of FBS. Cortices were then dissociated by mechanical trituration with glass pasteur pipettes. Dissociated cells were transferred in fresh culture medium (Neurobasal medium, Gibco), containing 2% B27 supplement (Gibco), 0.5 mM L-glutamine (200 Mm Gibco), and 1% penicillin/streptomycin (Gibco) and plated in 24- or 6-well plates previously coated with Poly-L-ornithin (0,1 mg/ml, Sigma-Aldrich) at a density of 250,000 cells/mL. Cultures at DIV5 were treated with recombinant aSyn (monomers and PFFs; final concentration of 100 nM) and long-term incubated until DIV25. No further media changes or addition of recombinant protein was made until the end of the culture. After mild trypsinization with 0.5% Trypsin–EDTA for 5 min to remove the excess of unbound material, cells were washed with PBS and fixed with 4% paraformaldehyde (PFA) for 20 min at RT. Fixed cultures were immunostained and subsequently analyzed. In addition, cells were lysed following the Triton soluble/insoluble biochemical assay described below. To verify the specificity of effects in our study, two batches of PFFs were used from two different labs. Protein concentration was estimated by the Bradford assay. Primary neuronal cultures were infected at DIV5 with the lentivirus pFUGW coding for WT aSyn or GFP.

### SDS-PAGE and Immunoblotting

After treatment, cells were washed with PBS and lysed on ice in radio-immunoprecipitation assay buffer (RIPA) (50 mM Tris pH 8.0, 150 mM NaCl, 0.1% Sodium-Dodecyl-Sulfate (SDS), 1% Nonidet P40, 0.5% Sodium-Deoxycholate, protease inhibitors, Roche). Lysates were centrifuged at 10,000 rpm and 4 °C for 10 min and post-nuclear supernatants were kept. Protein concentration was determined using the Bradford assay (Bio-Rad). All samples were measured in triplicate. For RIPA samples, equal protein amounts of denatured samples (5 min at 95 °C in 5 × protein sample buffer; 125 mM of 1 M Tris HCl pH 6.8, 4% SDS 0,5% Bromophenol blue, 4 mM EDTA 20% Glycerol 10% β-Mercaptoethanol) were subjected to SDS-PAGE on 12% separating gels with 7% stacking gels, using Tris–Glycine SDS 0.5% running buffer (250 mM Tris, 200 mM Glycine, 1% SDS, pH 8.3). The SDS insoluble fractions were denatured at 42 °C for 15 min. The transfer was carried out to 0.45 μm nitrocellulose membranes for 20 min per membrane at constant 25 mA in a semi-dry transfer chamber Trans-Blot® Turbo™ Transfer Solution from Bio-Rad (Bio-Rad). Membranes were blocked in 5% (w/v) skim milk (Fluka, Sigma-Aldrich) dissolved in 1 × TBS-Tween (50 mM Tris (hydroxymethyl)-aminomethane (TRIS) supplemented with 0.05% (v/v) Tween-20) for 1 h at RT. Incubation with the primary antibodies (anti-aSyn Syn-1 mouse, BD Transduction Laboratories 1:1000; anti-pS129-α-syn, Rabbit Abcam; mouse anti-*β*-actin, 1:10.000, Sigma) was performed overnight at 4 °C in 5% Albumin Bovine Fraction V (BSA)/TBS-Tween. Secondary antibodies (anti-mouse and anti-rabbit IgG, 1∶10,000 in TBS-Tween) were applied after three times washing in TBS-Tween, for 1 h at room temperature (RT). Membranes were visualized using Fusion Fx (Vilber Lourmat) with Immobilon Western Chemiluminescent HRP Substrate (Merck Millipore). Protein levels were quantified using ImageJ and normalized to the *β*-actin levels.

### Triton Soluble/Insoluble Biochemical Analysis

HEK293 cells were treated with aSyn monomers and PFFs for 48 h, harvested with trypsin, and washed with ice cold PBS and diluted 1:10 trypsin. The cell pellet was collected and solubilized in 1% triton buffer (50 mM tris pH 7.6, 150 mM NaCl, 2 mM EDTA, 1% triton, protease and phosphatase inhibitors), followed by 30-min incubation on ice. Cell lysates were then centrifuged at 13,000* g* for 30 min at 4 °C, and the supernatant was collected as the Triton soluble fraction. The pellet was washed with ice cold PBS. Insoluble fractions were prepared by resuspending the pellets in 2% SDS soluble buffer (50 mM tris pH 7.6, 150 mM NaCl, 2 mM EDTA, 2% SDS, protease and phosphatase inhibitors), sonicated and centrifuged at 13,000* g* for 10 min after incubation for 30 min at RT. The collected supernatant represents the SDS soluble fraction. The protein concentration was determined by the Bradford protein assay according to the manufacturer’s instructions, and the samples were then subjected to Western blot analysis. In order to estimate the effect of the levels of aSyn on seeding, we ran all samples in the same blot, or in pairs (HEK293-EGFP with HEK293-aSynEGFP, and HEK293 naive with HEK293-aSynBiFC).

### Filter Trap and Dot-Blot Assay

For the filter trap assay, equal protein amounts of recombinant aSyn monomers and PFFs were adjusted to equal volumes (diluted in PBS) and subsequently subjected to vacuum filtration through a 96-well dot blot apparatus (Bio-Rad) with an acetate cellulose membrane; immunoblotting (IB) was then performed with antibody against total aSyn (Syn-1). As a loading control experiment, the same samples were subjected to vacuum filtration through a 96-well dot blot apparatus with a nitrocellulose membrane followed by immunoblotting (IB) with Syn-1 antibody. Results shown are representative of 3 independent experiments.

### Mitochondrial Fractionation

Mitochondria were isolated as previously described [11], with some modifications. All buffers were prepared fresh before the isolation and placed in ice at least 15 min before harvesting the cells. Briefly, cells were harvested by trypsinization, pelleted and washed with cold-PBS buffer twice at 600 g for 5 min at RT. After the last wash, PBS was discarded and one volume of the cell pellet of IB 0.1 × buffer (NaCl 2.5 mM, MgCl_2_ 0.5 mM, TRIS 3.5 mM pH = 7.8) was added. The cells were resuspended gently and homogenized with 15 strokes using a Heidolph D-91126 Type: RZR1 homogenizer at 1500 rpm. The sample was transferred to Eppendorf 1.5-mL tubes and 1/10 of the initial cell pellet volume of IB 10 × Buffer (NaCl 0.25 M, MgCl_2_ 50 mM, TRIS 0.35 M pH = 7.8) was added. A microcentrifuge was used to pellet the debris, unbroken cells, and nuclei at 1200 rpm for 3 min at 4 °C. The supernatant that contained mitochondria and cytoplasmic fraction was collected. An additional centrifuge step was performed at 1200* g* for 3 min at 4 °C to obtain a cleaner fraction and avoid contamination of “heavy material.” The supernatant was collected again. Finally, the collected supernatant was centrifuged at 1500 rpm for 2 min at 4 °C. The obtained pellet was the mitochondrial fraction and the supernatant was the cytoplasmic fraction, respectively. After collection of the cytoplasmic fraction, the mitochondrial fraction was resuspended in RIPA buffer. Protein concentration was determined by the Bradford protein assay and the samples were then subjected to Western blot analysis.

### Immunocytochemistry

For ICC, cells were washed with PBS and diluted 1:10 Trypsin, fixed with 4% paraformaldehyde (PFA) for 20 min at RT, and followed by a permeabilization step with 0.5% Triton X-100 (Sigma-Aldrich) for 20 min at RT. After blocking in 1.5% normal goat serum (PAA)/DPBS for 1 h, cells were incubated with primary antibody. Primary antibodies used were as follows: anti-aSyn Syn-1 mouse, BD Transduction Laboratories (1:1000); anti-pS129-α-syn Rabbit Abcam (1:1000), incubated overnight at 4 °C and secondary antibody (Alexa Fluor 488 donkey anti-mouse IgG and/or Alexa Fluor 555 goat anti rabbit IgG, (Life Technologies- Invitrogen, Carlsbad, CA, USA) for 2 h at RT. Nuclei were stained with 4′6′-diamidino-2-phenylindol (DAPI, Sigma-Aldrich) (1∶5000 in DPBS) for 10 min. After a final wash, coverslips were mounted by using Mowiol (Sigma-Aldrich) and subjected to fluorescence microscopy. Images were analyzed using LAS AF v.2.2.1 (Leica Microsystems) software.

### RT-QuIC Assay

Real-time quaking-induced conversion (RT-QuIC) was performed as a modification of previously proposed methods for different amyloid proteins [12, 13] and optimized in our lab. In brief, a reaction mixture with the following composition was made: 150 mM NaCl, 1 mM EDTA, 10 μM ThioT, 70 μM SDS, 10 μg/ml of monomeric aSyn and fibrillar aSyn [6] at a final concentration of 0.1 μg/ml, 1 μg/ml, 10 μg/ml and 100 μg/ml in PBS buffer. A total of 100 μl of this reaction mixture was pipetted in black 96-well plates (Corning Incorporated, WA, USA) in triplicates. Additionally, cell lysates were added to the mixture to a final protein concentration of 0.1 µM. Finally, the plates were covered with sealing tape and incubated in a plate reader (41 °C, Infinite M200 fluorescence plate reader, Tecan, Hamburg, Germany) for 250 amplification cycles (1-min orbital shaking at 432 rpm; 2-min incubation; measurement of fluorescence intensity at 480 nm). Endpoint fluorescent intensities were normalized by baseline values.

### Statistical Analyses

Statistical analyses were performed using the Student’s *t*-test or 2-way ANOVA for comparisons of independent variables. The data are presented as mean ± standard deviations and represent results from at least 3 independent experiments. Differences were considered statistically significant when *p < 0.05, **p < 0.01, and ***p < 0.001.

## Results

### Exogenous aSyn Preformed Fibrils (PFFs) Induce aSyn Aggregation and Accumulation of Endogenous Phosphorylated pS129aSyn Inclusions

**Fig. 1:**
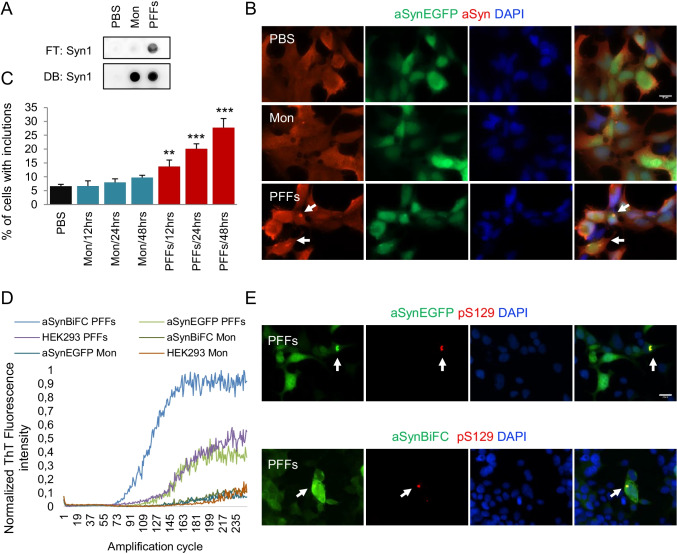
Exogenous aSyn preformed fibrils (PFFs) induce aSyn aggregation. **A** Filter trap and dot blot assay from recombinant (100 nM) aSyn both diluted in PBS. **B** Indicative immunofluorescence of the HEK293-aSynEGFP stable cell line stained with anti-aSyn antibody, after treatment with aSyn monomers and PFFs (100 nM/48 h) shows to formation of aSyn aggregates after treatment with PFFs (Scale bar 25 μm). **C** Quantification of the cells with aSyn inclusions shows a progressive formation of inclusions over time in cells treated with PFFs for 48 h (*n* = 4, mean ± SD). **D** RT-QuIC from cell lysates under our assay conditions reveals a higher aSyn aggregation after treatment with aSyn PFFs, as demonstrated by the increased value of ThT-fluorescence under the different concentrations. Graphs show the mean value from four independent experiments. **E** Fixed HEK293-aSynBiFC and HEK293-aSynEGFP cells treated with aSyn PFFs immunostaining with an anti-p-Ser129 (red) antibody. DAPI was used for nuclear staining (Scale bar 25 µm)

The overall objective of our study was to investigate the effect of extracellular aSyn PFFs on the seeding and aggregation of endogenous aSyn expressed at variable levels in cells. For this purpose, we employed simple but highly tractable cellular bioreactors (stable human embryonic kidney (HEK293) cells) expressing aSyn fused with EGFP (HEK293-aSynEGFP), to assess the effects in living cells. Individual clones from both stable cell lines were selected for our experiments. Cells were treated with exogenous aSyn monomers or PFFs, characterized by immunoblot and electron microscopy before and after sonication (Supplementary Fig. 1A and B) [6], at a final concentration of 100 nM, for 48 h. Control cells were exposed to vehicle only (PBS). We also quality-checked aSyn monomers and fibrils using a filter trap assay (Fig. 1A). To ensure we loaded comparable amounts of protein, a dot-blot was run in parallel with the same samples. Immunofluorescence analyses of treated cells showed that PFFs induced the formation of aSyn inclusions that were EGFP-positive, confirming the seeding of the endogenously expressed aSynEGFP by the PFFs (Fig. 1B), as this was not observed in PBS-treated cells. Quantification of the aSynEGFP inclusions revealed a progressive and time-dependent process that resulted in almost 25% of the cells displaying aSyn inclusions (Fig. 1C). No aggregates were detected after treatment with 100 nM of aSyn monomers.

To further validate our results and assess reproducibility between different sources of aSyn, we repeated the experiments with aSyn monomers and PFFs prepared independently, in a different laboratory (Mon2, PFFs2, Supplementary Fig. 1C). We found no differences between the results obtained with the two different sources PFFs (Supplementary Fig. 1C). All experiments included in the subsequent experiments were performed using PFFs1.

Next, we used the real-time quaking-induced conversion (RT-QuIC) assay, an ultrasensitive biochemical assay based on the binding of thioflavin T (ThT) by amyloid fibrils, to estimate the seeding activity of aSyn [14–16]. We used the HEK293-aSynEGFP cells and a stable cell line reporting on aSyn oligomerization based on the bimolecular fluorescence complementation (BiFC) assay HEK293-aSynBiFC [17, 18] (Supplementary Fig. 2). RT-QuIC from the different cell lines non-treated with aSyn monomers and PFFs (*n* = 4) showed lower ThT fluorescence intensity than that of cells treated with monomers and PFFs (Supplementary Fig. 2A). We verified that the addition of PFFs induced seeding of monomeric aSyn leading to an increase in the overall normalized ThT fluorescence levels consisted with higher aggregation levels [6] (Supplementary Fig. 2B). Importantly, the initial ThT fluorescence intensity values were not significantly different among groups of the different cell lines, suggesting the absence of additional unbound PFFs in the reaction buffer (Supplementary Fig. 2C). Addition of PFFs also led to shorter lag times, i.e., earlier and faster aggregation, consistent with the seeding effect (Supplementary Fig. 3D). As expected, higher concentrations of PFFs resulted in shorter lag phases and higher overall normalized ThT fluorescence levels. Next, cells were incubated with aSyn monomers or PFFs and reaction mixtures of cell lysates to final protein concentrations of 100 nM and were tested using the RT-QuIC assay. Interestingly, we observed stronger aSyn aggregation with cell lysates followed by treatment with aSyn PFFs, as demonstrated by the increase in ThT signal with the different concentrations. Importantly, the kinetics of conversion was identical between the cell lines seeded with cell lysates prepared after treatment with PFFs, unlike those seeded with lysates prepared from cells treated with aSyn monomers, with almost no amplification (Fig. 1D and Supplementary Fig. 2D). In addition, immunostaining experiments against Serine 129 confirmed the accumulation of endogenous phosphorylated pS129aSyn inclusions in both HEK293-aSynBiFC and HEK293-aSynEGFP cell lines (Fig. 1E). In contrast, treatment with aSyn monomers did not induce the accumulation of pS129aSyn (Supplementary Fig. 3A and B). Overall, we concluded that addition of aSyn PFFs induced aSyn seeding and accumulation of pS129aSyn aggregates. These data provide evidence that the extent to which endogenous aSyn aggregates depends on the expression levels of aSyn.

### Different Expression Levels of aSyn Define the Seeding of Exogenously Added aSyn PFF Seeds

**Fig. 2:**
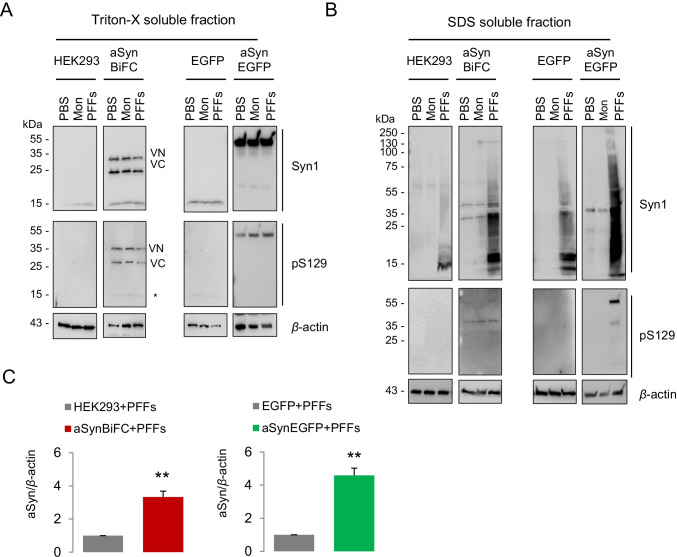
aSyn fibrils seed endogenous aSyn to different extent depending on the endogenous levels. **A, B** Immunoblot for the Triton-X soluble and SDS-soluble samples extracted from the different cell lines (HEK293, HEK293-aSynBiFC, HEK293-EGFP, and HEK293-aSynEGFP) treated with 100 nM of aSyn monomers and PFFs diluted in PBS. Cells treated with PBS were used as the control. Immunoblot analysis against pS129 and Syn-1 resulted in a shift of the SDS-soluble aSyn in higher molecular weight species. This SDS-soluble aSyn fraction was significantly enriched in the cell lines overexpressing aSyn (HEK293-aSynBiFC and HEK293-aSynEGFP) compared to the control cell lines (HEK293 and HEK293-EGFP, respectively). High molecular weight species with aSyn PFFs were also positive for the phospho Ser 129 aSyn antibody. *β*-Actin was used as a loading control. **C** Densitometry of the SDS soluble fractions confirmed the significant difference between the cell lines (*n* = 3, mean ± SD)

Focusing on the comparative analysis of the seeding effect between the different cell lines, we examined whether the different expression levels of aSyn modulated the uptake and seeding of exogenously-added aSyn PFFs. Therefore, cell lysates from the different cell lines were fractionated using Triton-X- and SDS-containing buffers. The seeding capacity of HEK293-aSynBiFC and HEK293-aSynEGFP stable cell lines was compared with that of naïve HEK293 and HEK293-EGFP cell lines. We found that high molecular weight (HMW) aSyn species were detected in PFF-treated cells in the SDS-soluble fraction in both stable cell lines expressing aSyn, whereas mostly monomeric species were detected in the Triton X-100 soluble fraction (Fig. 2A). The comparison between the HEK293-EGFP stable cell line and the HEK293-aSynEGFP line revealed, as expected, higher levels of aSyn in the later cells, demonstrating the contribution of the endogenous levels towards seeding. In addition, we detected an increase in phosphorylated aSyn on S129 in the SDS soluble fraction (Fig. 2B), in agreement with the immunostaining results (Fig. 1E). Consistently, we observed identical effects when comparing the HEK293-aSynBiFC cell line with naïve HEK293 cells (Fig. 2C and Supplementary Fig. 4A and B).

Overall, we demonstrate that there is a clear correlation between the endogenous of aSyn and the seeding efficiency followed by the accumulation of extracellular pathological assemblies.

### Treatment with aSyn PFFs Results to Accumulation of Truncated aSyn in Mitochondria

**Fig. 3:**
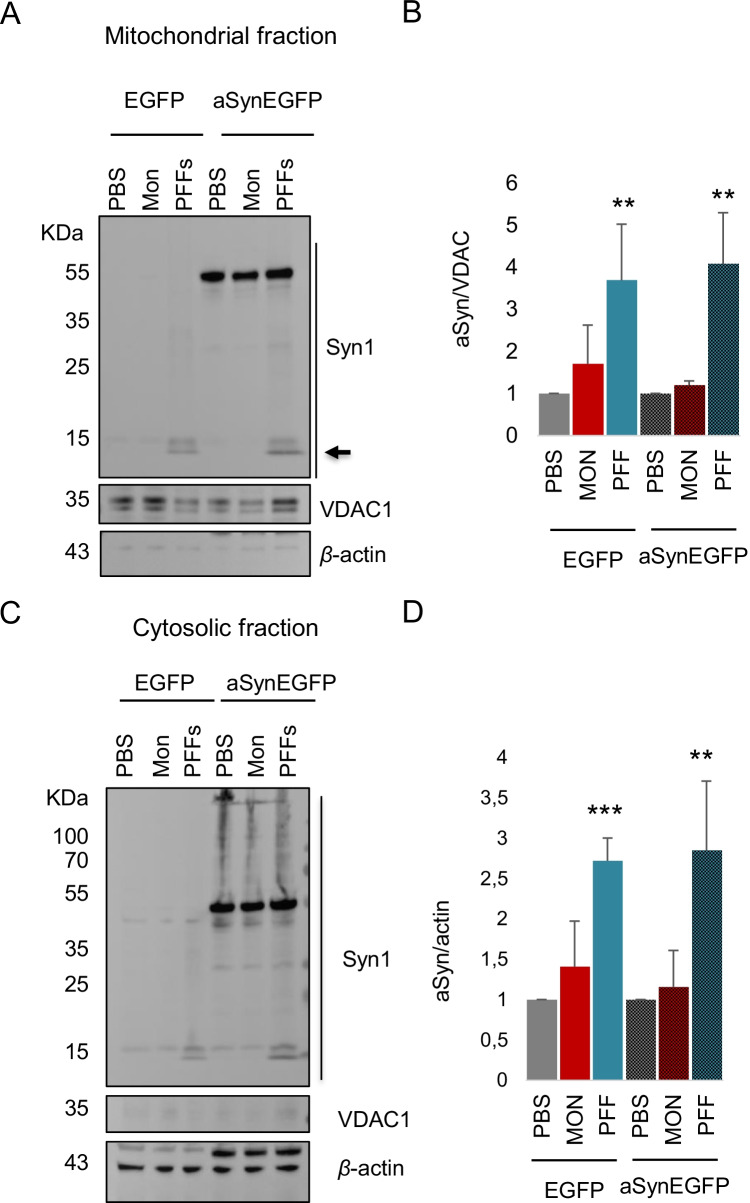
Treatment with aSyn fibrils induces accumulation of truncated aSyn in mitochondria. **A** Immunoblot of the mitochondrial extracts from HEK293-EGFP and HEK293-aSynEGFP stable cell lines shows increased localization of aSyn truncated (indicated with arrow) after treatment with aSyn PFFs. VDAC was used as a loading control. **B** Densitometric analysis of fractionated mitochondrial lysates (*n* = 3, mean ± SD). **C** Immunoblot of the cytosolic extracts from HEK293-EGFP and HEK293-aSynEGFP stable cell lines. *β*-Actin was used as a loading control. **D** Densitometric analysis of fractionated cytoplasmic lysates (*n* = 3, mean ± SD)

Mitochondrial dysfunction has long been implicated in PD, and several studies suggest that aSyn may associate with mitochondria and with mitochondrial proteins [9, 19, 20]. Furthermore, pathogenic phospho-aSyn aggregates bind to mitochondria leading to cellular respiration defects [21]. Therefore, we examined whether aSyn could be detected in mitochondria, and whether the localization of aSyn in mitochondria was altered upon treatment with aSyn monomers or PFFs. Using subcellular fractionation, and using VDAC1 as a mitochondrial marker and *β*-actin as a cytosolic marker, we confirmed the presence of aSyn in the mitochondrial fraction. As expected, we detected a strong increase of aSynEGFP in the mitochondrial fraction upon overexpression. Furthermore, we observed a robust increase of truncated aSyn, mostly after treatment with aSyn PFFs (Fig. 3A–D). Treatment with PFFs promoted the localization of aSyn in the mitochondrial fraction in both HEK293-aSynEGFP and HEK293-EGFP cells. Surprisingly, no obvious difference was observed on the accumulation of aSyn in the mitochondria in cells expressing aSynGFP comparing to cells expressing only GFP (Fig. 3B). In addition, we examined if treatment with aSyn PFFs affected the levels of mitochondrial proteins such as OPA1, DRP1, HSP27, Mitofusin, and TRAP1. Immunoblot analyses showed that increased localization of aSyn in mitochondria did not alter the levels of mitochondrial proteins (Supplementary Fig. 5A and B).

### Increased Levels of aSyn Enable Accelerated Seeding by aSyn PFFs in Primary Neuronal Cultures

**Fig. 4:**
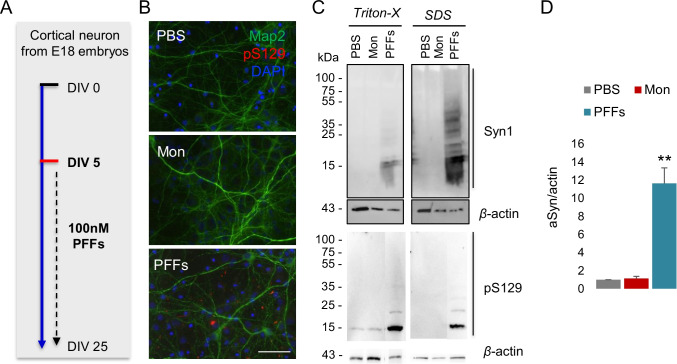
aSyn fibrils seed endogenous aSyn in primary neuronal cultures. **A** Seeding model in primary cortical neurons. At DIV5 aSyn, monomers and PFFS were added and incubated until DIV25, when neurons fixed and counterstained with Map2 (green) and pS129 (red) antibodies. **B** Representative immunofluorescence showing the formation of phospho-Ser129 only at PFF-treated neurons for the indicated time. DAPI was used for nuclear staining (Scale bar 50 μm). **C** Immunoblot for the Triton-X soluble and SDS-soluble samples extracted from DIV25 showing that aSyn high molecular weight (HMW) species were enriched in SDS-soluble fraction, as well as the detection of pS129 aSyn after treatment with PFFs. *β*-Actin was used as a loading control. **D** Densitometry of the SDS-soluble fractions shows the significant increase of the HMW species under PFFs treatment (*n* = 3, mean ± SD)

Next, we examined the seeding effect of aSyn PFFs in primary cortical neurons. Briefly, we treated primary neurons at DIV5 with aSyn monomers or PFFs and incubated the cultures until DIV25 (Fig. 4A). Using immunocytochemistry, we detected the accumulation of pS129aSyn upon treatment with PFFs (Fig. 4B). Consistently, we observed the presence of HMWs of aSyn and increased accumulation of pS129aSyn in the SDS-soluble fraction, particularly after treatment with aSyn PFFs (Fig. 4C and D).

**Fig. 5:**
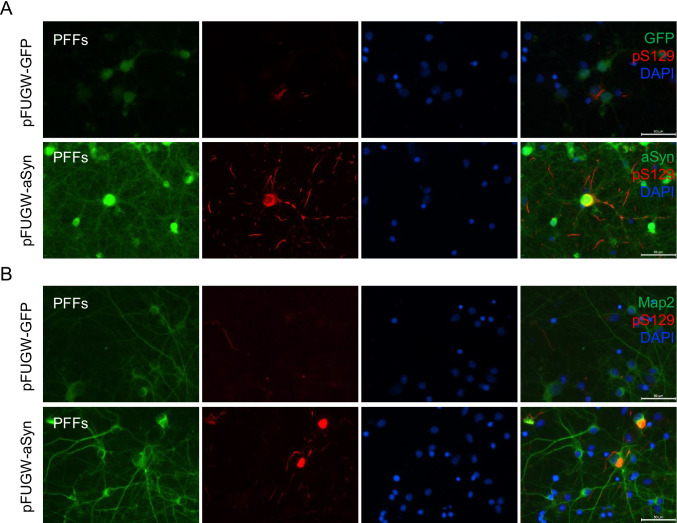
aSyn overexpression in primary cortical neurons results to a robust increase of pS129 under treatment with aSyn PFFs. **A** Infection of primary cortical neurons at DIV5 with lentiviral vector for aSyn WT (pFUGW-aSyn). As a control, cells were infected with viruses encoding GFP (pFUGW-GFP). The same day, aSyn monomers and PFFs were added and incubated till DIV25, were neurons fixed and counterstained with aSyn (green) and pS129 (red) antibodies. For the pFUGW-GFP neurons, we checked the GFP fluorescence (green) and stained for pS129 (red). Indicative immunofluorescence showing the formation of phospho-Ser129 only at PFF-treated neurons for the indicated time. DAPI was used for nuclear staining (Scale bar 50 μm). **B** Indicative immunofluorescence of neurons counterstained with Map2 (green) and pS129 (red) antibodies. DAPI was used for nuclear staining (Scale bar 50 μm)

Finally, we assessed the seeding of exogenous aSyn in primary cortical neurons on the seeding by exogenous aSyn seeds in primary cortical neurons transduced with lentiviral vectors encoding aSyn or GFP as a control (Fig. 5A and B). Interestingly, we found increased accumulation of phosphorylated pS129aSyn aggregates in aSyn WT-infected neurons at DIV25. This was absent in cells treated with aSyn monomers or with PBS (Supplementary Fig. 6 and 7). On the other hand, we observed lower pS129aSyn accumulation in GFP-infected cells, similar to what was observed with non-infected cells, indicating that phosphorylation of aSyn at Ser129 depends on endogenous aSyn levels. These data are in line with those observed using stable cell lines and demonstrate that the levels of aSyn influence the extent of seeding and the formation of pathological aSyn assemblies.

## Discussion

Duplications or triplications of the *SNCA* gene are associated with early onset familial forms of PD, as well as motor and cognitive dysfunction [22, 23]. In fact, there is a clear dosage effect showing that an increase in *SNCA* copies leads to earlier disease onset and faster progression [23–25].

Here, we used simple cellular models to investigate the relationship between the cellular uptake of exogenously-applied aSyn PFFs and the pathological conversion of endogenously expressed of aSyn into aggregated species. We observed an increased percentage of cells with inclusions and more insoluble aSyn in cells expressing aSynEGFP. Also, we detected an increase in phosphorylated aSyn in the SDS-soluble fraction, confirming seeded aggregation. Similar results were obtained when we used aSynBiFC cells. However, the levels of pS129 aSyn aggregates were lower in the later cells. Given the nature of the models, this limited detection of pS129 aSyn inclusions in the aSynBiFC cells suggests that aggregation precedes the pathological accumulation of pS129 aSyn deposits, as previously reported using an inducible model of seeding in SH-SY5Y cells. In this study, the formation of insoluble pS129 aSyn inclusions was observed when a greater amount of fibrils was applied to the cells, and for a longer incubation period [26]. Consistently, we found that using a lower amount of PFF seeds rarely resulted in the detection of pS129 aSyn-positive aggregates (data not shown). Using the RT-QuIC assay, we detected seeding in reactions where the cell lines were treated with aSyn PFFs but not in reactions where the cell lines were treated with monomeric aSyn. In accord with recent literature, the mitochondria of HEK293-aSynEGFP cells were enriched in aSynEGFP when compared to those from control HEK293-EGFP cells [27, 28]. In addition, fractionation of mitochondrial and cytosolic extracts revealed the presence of aSyn in mitochondria, mostly as truncated forms. These data demonstrate that differences in the endogenous levels of aSyn may render the cells more susceptible to the seeding effects of aggregated (PFFs) aSyn. This is consistent with a recent study showing that the accumulation of aSyn significantly differed between primary neuronal cultures from various brain regions characterized by different expression levels of aSyn [29].

Primary hippocampal neuronal cultures exhibit different aSyn expression profiles among cells since these cultures are composed of several neuronal and non-neuronal cells [30]. In this study, the authors demonstrate that the subpopulation of cells with higher intracellular levels of aSyn is more prone to seeding, exhibiting the highest pathology and selective vulnerability [30]. This is further supported by the fact that abundant aSyn expression levels observed in neurons where brain regions were affected at early PD stages [31, 32], reinforcing the hypothesis that an increase in aSyn expression may be a risk factor for neurodegeneration. Thus, the heterogeneity of endogenous aSyn levels is reflected in selective vulnerability among neuronal cells and provides an explanation for the selective vulnerability of the brain regions observed in synucleinopathies. Consistently, we found that overexpression of aSyn in primary neuronal cultures results in accelerated accumulation of pS129aSyn aggregates after exposure to PFFs.

Collectively, our study is consistent with the possibility that brain regions expressing higher levels of aSyn may contribute more strongly to the spreading of pathology. Alternatively, it could also be that, in such brain areas, the balance between aSyn S129 phosphorylation/dephosphorylation is shifted towards the earlier, thereby leading to the accumulation of higher levels of aSyn phosphorylated on S129.

In the future, a better understanding of the expression profile of aSyn will be important to better understand the physiological function of aSyn as well as the implicated mechanisms involved in pathological aggregation. Ultimately, it will also be important to understand how the levels of aSyn vary in different subcellular compartments and what pathological alterations (e.g., S129 phosphorylation) take place in such compartments (e.g., in the nuclei or mitochondria).

## Conclusions

In conclusion, by using tractable stable cell lines and primary neuronal cultures with different expression levels of aSyn, we demonstrate that the endogenous aSyn levels are a key element that defines the seeding by extracellular aSyn PFFs. Our study puts forward models that will now be useful for addressing additional molecular mechanisms regulating the spreading and pathological accumulation of aSyn and for the identification of novel targets for therapeutic intervention.

## Supplementary Information

Below is the link to the electronic supplementary material.

Supplementary file1 Supplementary Figure 1. Exogenous aSyn PFFs induce aSyn aggregation. Comparison between different PFFs. A. Representative SDS-PAGE image of the recombinant proteins used for further experiments. Western blot analysis of 100ng re-combinant aSyn proteins (monomers and PFFs) loaded in SDS-PAGE Gel 12%. B. Electron micrographs of PFFs before (A) and after (B) sonication (Scale bars 500 nm) [6]. C. Treat-ment with recombinant aSyn from different origin and quantification of the percentage of cells with aSyn inclusions. For these experiments we treated the cell with recombinant aSyn produced in the laboratory of Dr. Melki (PFFs 1= our own material and PFFs 2 = Melki’s material; n=3, mean ± SD). Production of human recombinant monomeric WT aSyn and fibrils assembly was prepared as previously described [33]. Supplementary Figure 2. RT-QuIC control experiments using different amounts of PFFs. A. RT-QuIC amplification of aSyn in the presence of different cell lines non-treated with aSyn monomers and PFFs (n=4). Normalized ThT fluorescence intensity is generally lower than that for cells treated with monomers and PFFs. B. Effect of different PFFs quantities in the RT-QuiC amplification of 1 µg of monomeric aSyn. Higher PFFs quantity leads to shorter lag times and higher overall normalized ThT fluorescence levels (n=3). C. Initial normalized ThT fluorescence intensity values among cell lines shows no significant differences between the different groups (n=4). D. RT-QuIC from the different cell lines treated with aSyn monomers and PFFs, showing all the additional controls for the reaction (n=4). Supplementary Figure 3. Representative images from fixed HEK293-aSynEGFP (A) and HEK293-aSynBiFC (B) cells after treatment with PBS, aSyn monomers and PFFs. Immunostaining with an anti-p-Ser129 (red) antibody. DAPI was used for nuclear staining (Scale bar 25 µm). Supplementary Figure 4. Representative immunoblot showing the expression levels of aSyn from the HEK293-aSynEGFP and HEK293-aSynBiFC stable cell lines. A. SDS-PAGE with RIPA extracts showing the different expression levels of aSyn among the different cell models. B. Densitometry of the SDS soluble fractions from the different stable cell lines confirmed the significant difference between the cell lines (n=3, mean ± SD). Supplementary Figure 5. Expression levels of mitochondrial proteins under treatment with αSyn PFFs. A, B. Immunoblot of the mitochondrial extracts from HEK293-EGFP and HEK293-aSynEGFP stable cell lines  for the proteins OPA1, DRP1, HSP27, Mitofusin and TRAP1.  VDAC was used as a loading control for the mitochondrial extracts and β-actin for the cytosolic respectively (n=3, 2way ANOVA – Dunnett’s multiple comparisons Test. Adjusted P value *<0,05, **<0,01). Supplementary Figure 6. Representative images from fixed primary neuronal cultures infected with lentiviral vector expresses aSyn under treatment with PBS, aSyn monomers and PFFs. A. Primary neuronal cultures infected with pFUGW-EGFP. Immunostaining with an anti-p-Ser129 (red) antibody . DAPI was used for nuclear staining (Scale bar 50 µm). B. Primary neuronal cultures infected with pFUGW-aSyn. Immunostaining with an anti-p-Ser129 (red) and Syn1 (green) antibodies. DAPI was used for nuclear staining (Scale bar 50 µm). Supplementary Figure 7. Representative images from fixed primary neuronal cultures infected with lentiviral vector expresses aSyn under treatment with PBS, aSyn monomers and PFFs. A, B. Primary neuronal cultures infected with pFUGW-EGFP and pFUGW-aSyn respectively. Immunostaining with an anti-p-Ser129 (red) antibody and Map2 (green) antibodies. DAPI was used for nuclear staining (Scale bar 50 µm) (PDF 1148 KB)

## Data Availability

The data that support the findings of this study are available from the corresponding author upon reasonable request.

## Abbreviations

aSyn: Alpha-synuclein
DAPI: 4′6′-Diamidino-2-phenylindol
Dox: Doxycycline
HMWs: High molecular weights species
LBs: Lewy bodies
LNs: Lewy neurites
PD: Parkinson’s disease
PFFs: Preformed fibrils
pSyn: P-Ser129 alpha-synuclein
RT-QuIC: Real-time quaking-induced conversion
ThT: Thioflavin T
WT: Wild type
