# Supplementary material for: Endogenous Levels of Alpha-Synuclein Modulate Seeding and Aggregation in Cultured Cells
Source: Mol Neurobiol. 2022 Jan 4;59(2):1273–84. doi: 10.1007/s12035-021-02713-2 (PMC8857012; doi:10.1007/s12035-021-02713-2)

Supplementary Figure 1

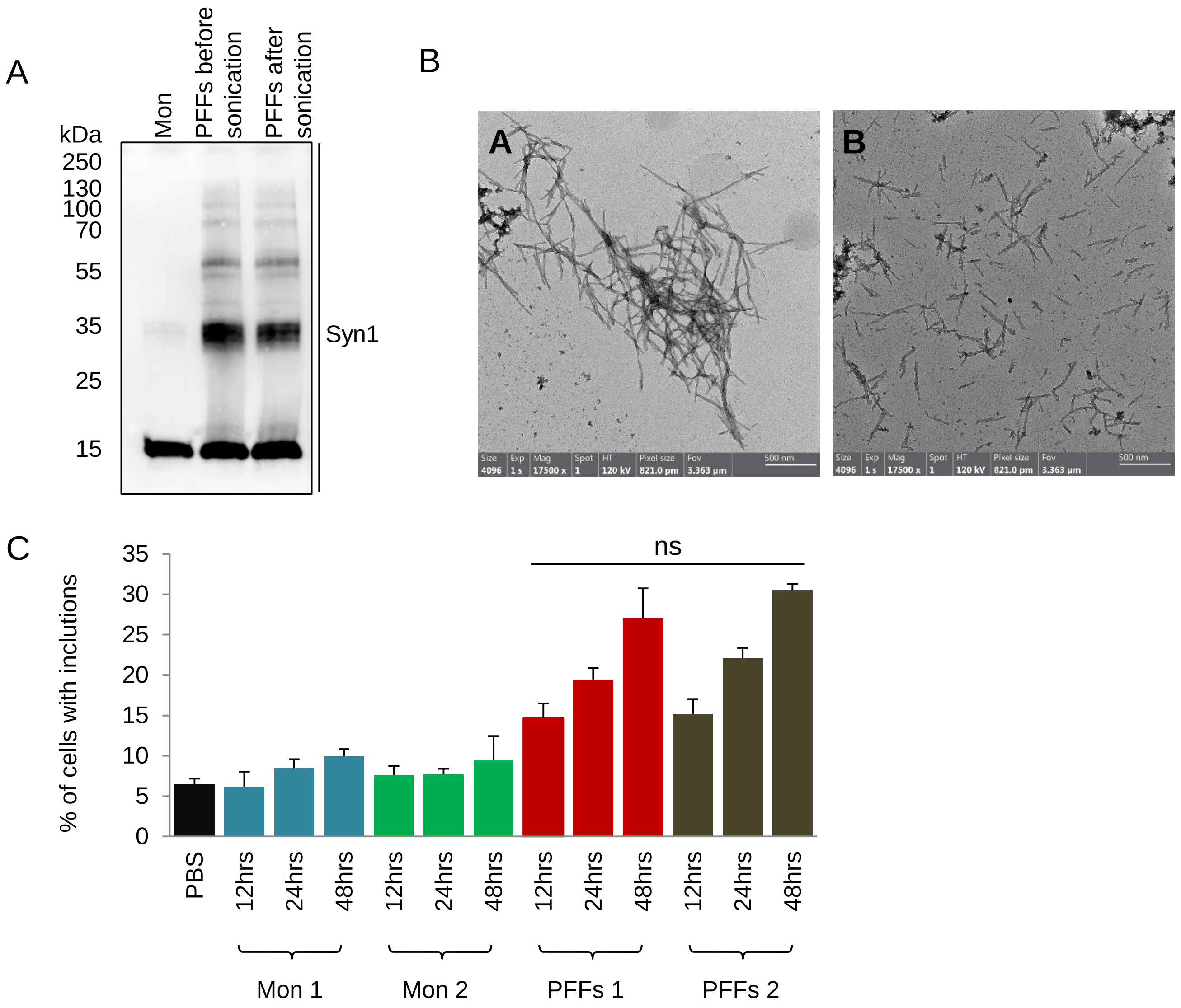

Supplementary Figure 2

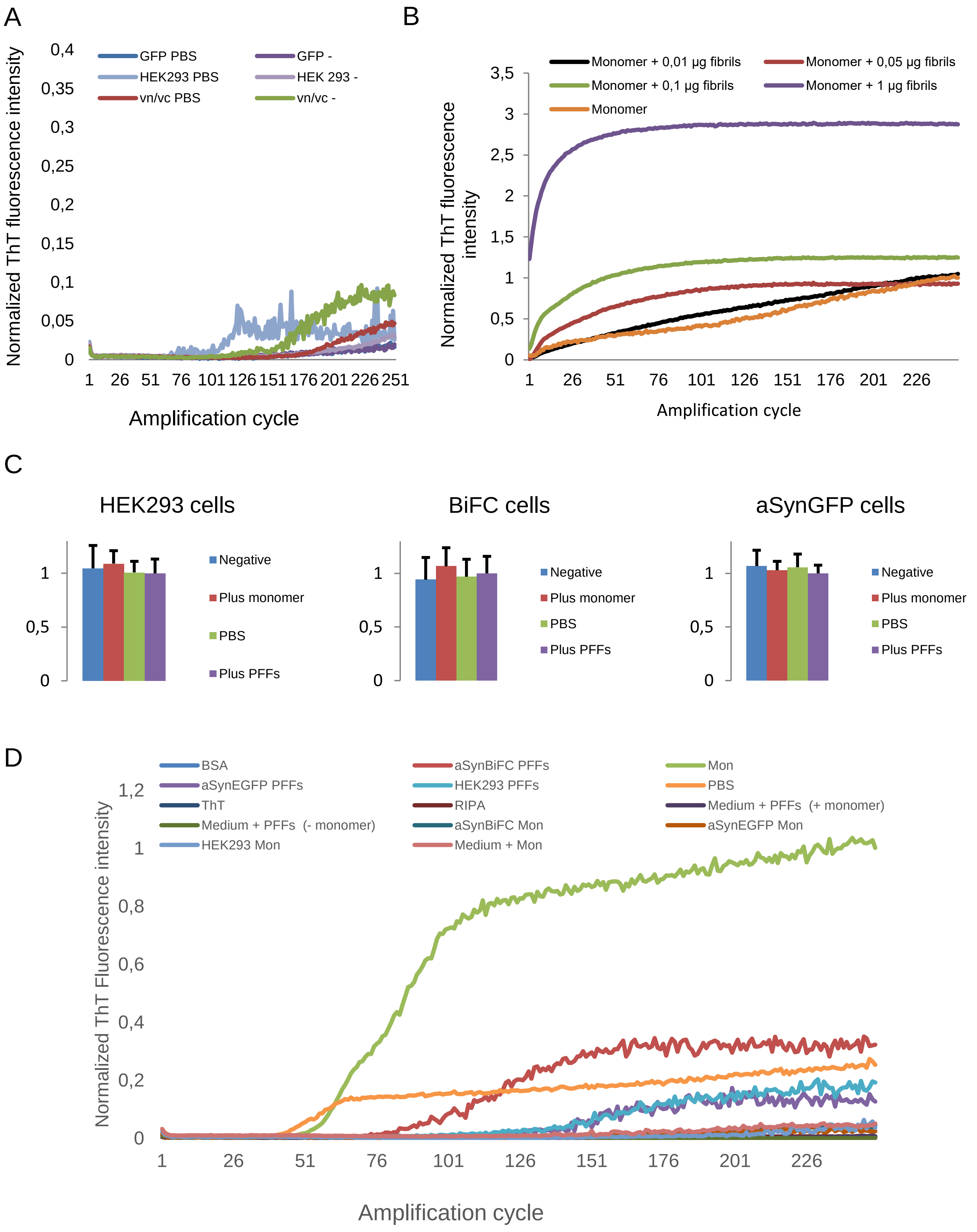

Supplementary Figure 3

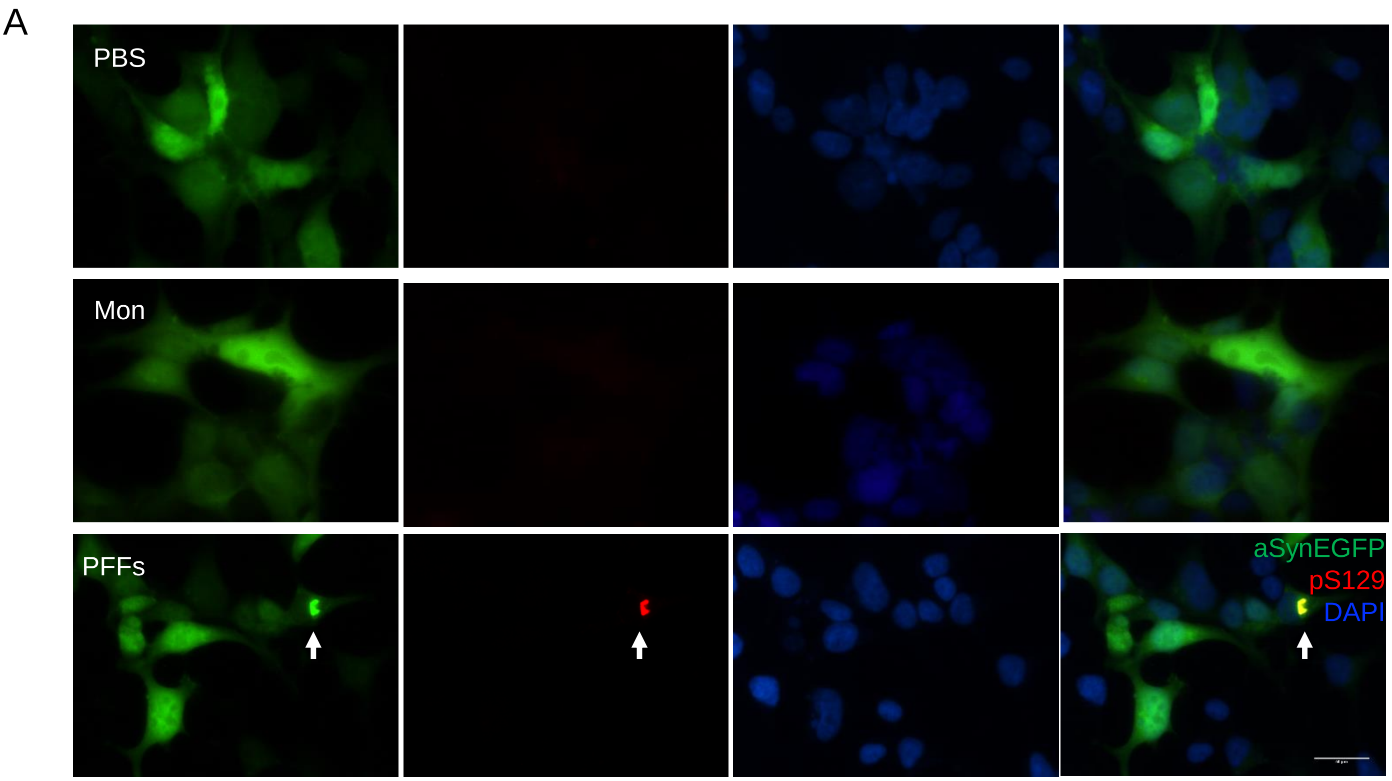

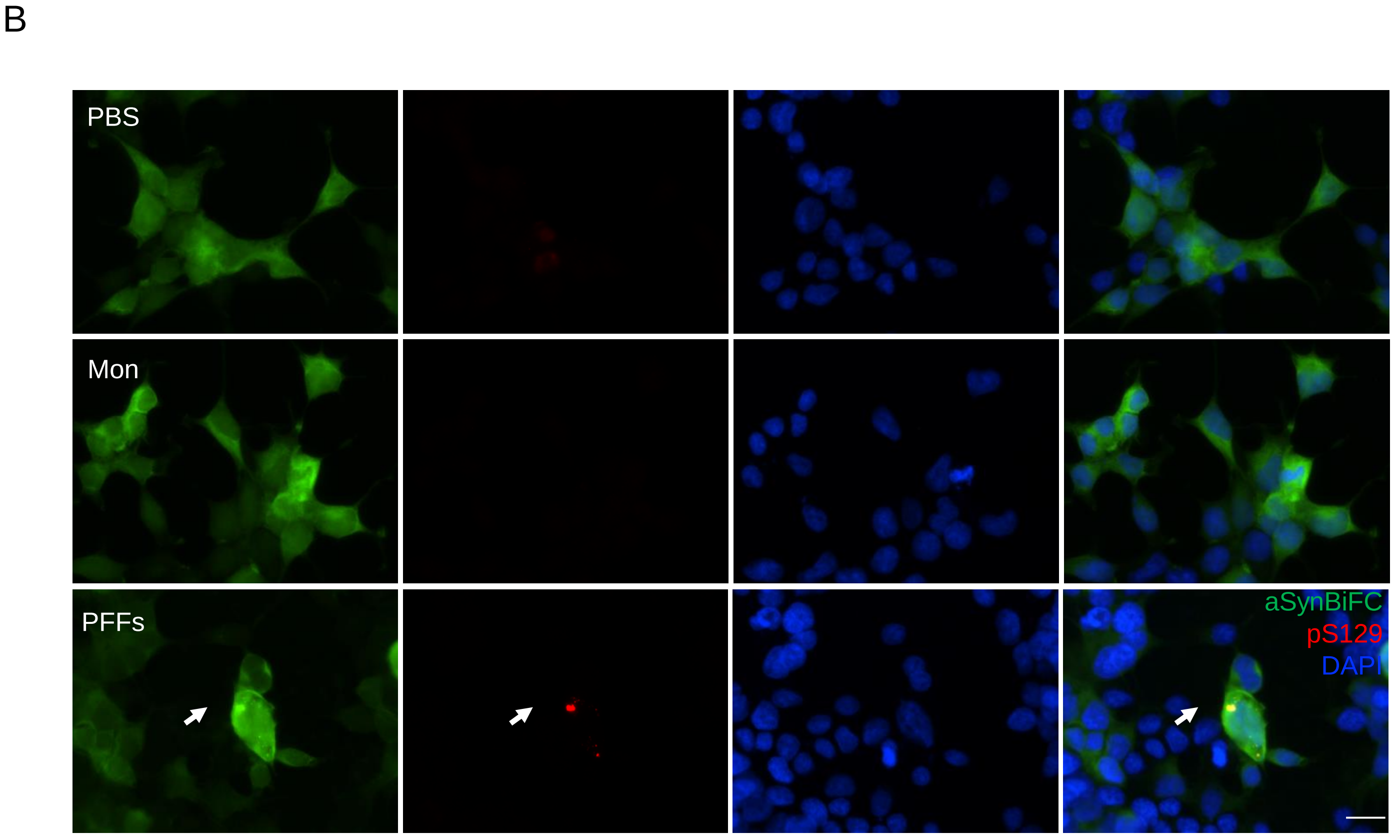

Supplementary Figure 4

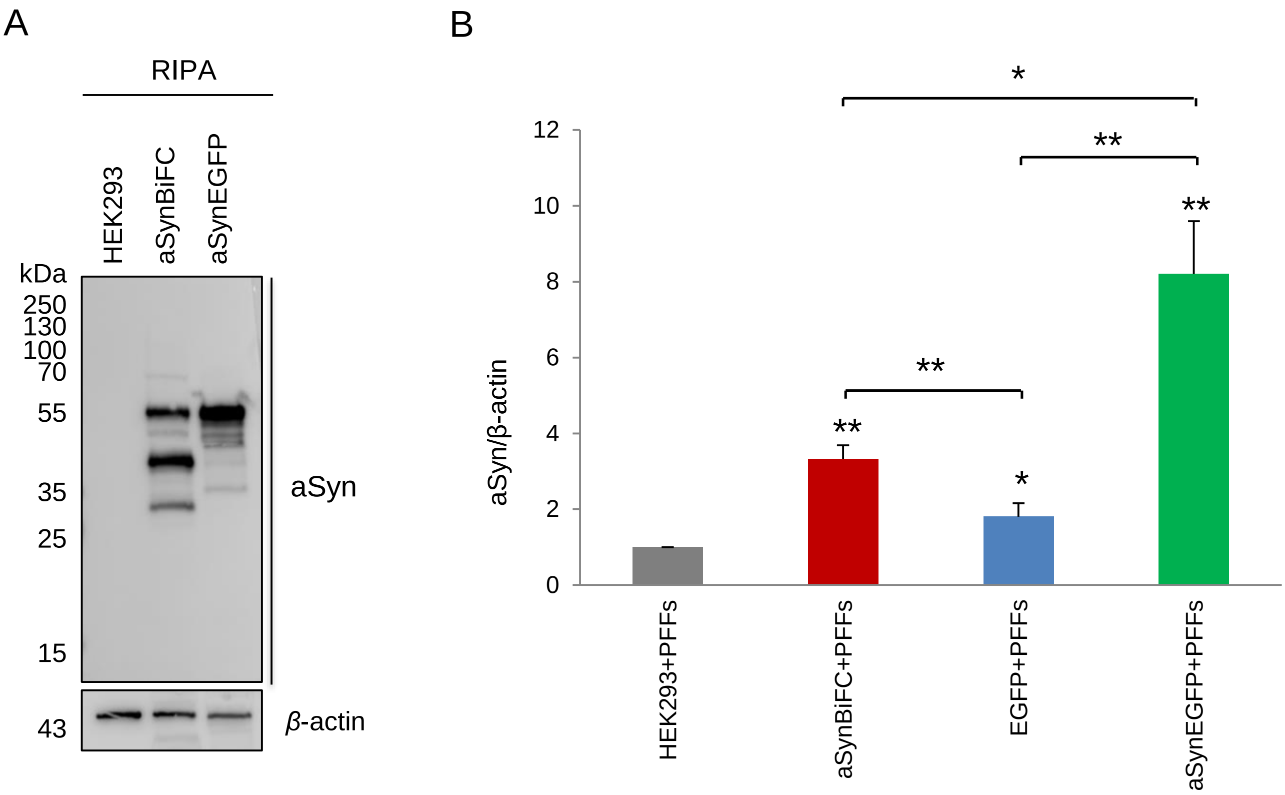

Supplementary Figure 5

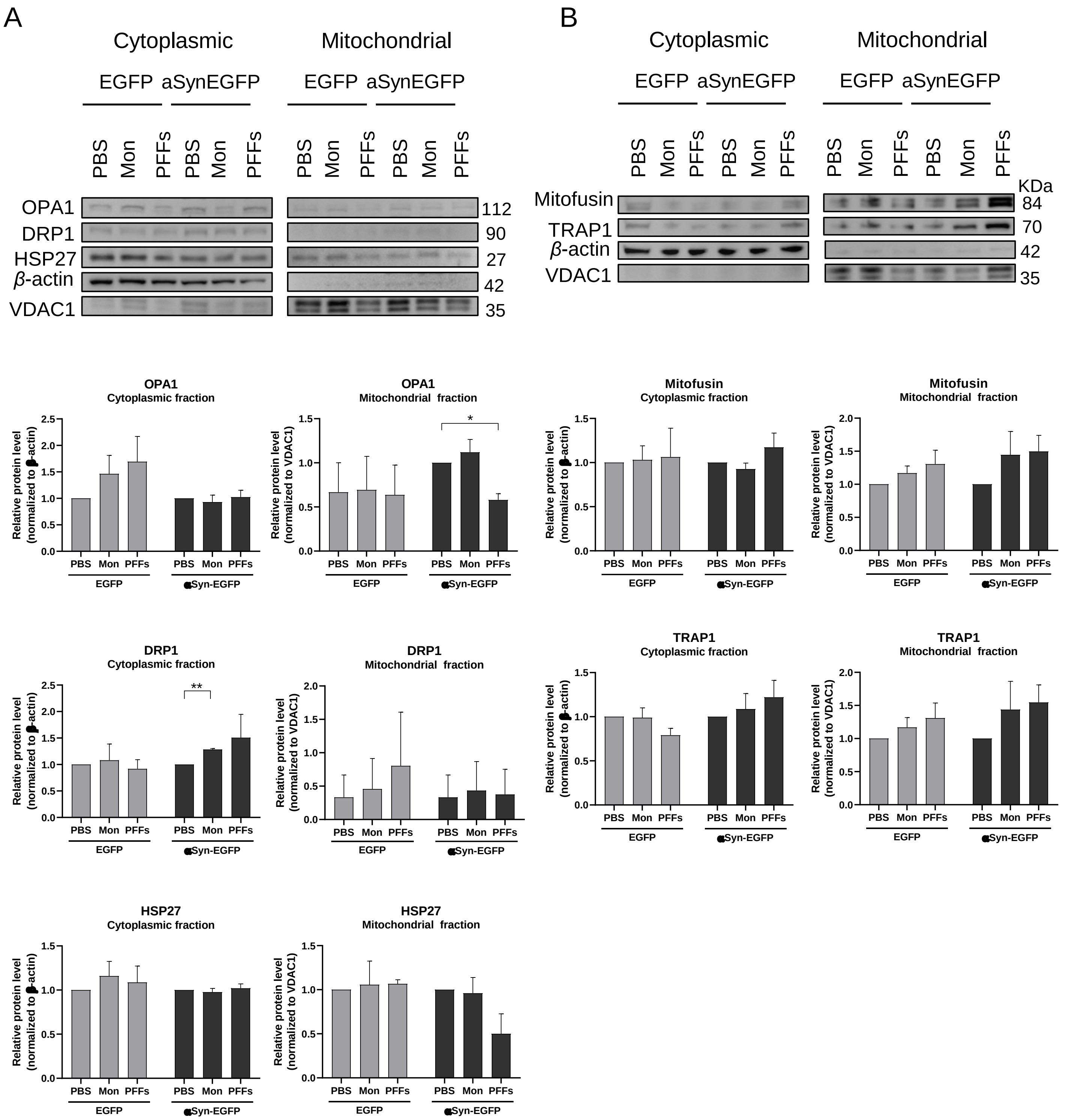

Supplementary Figure 6

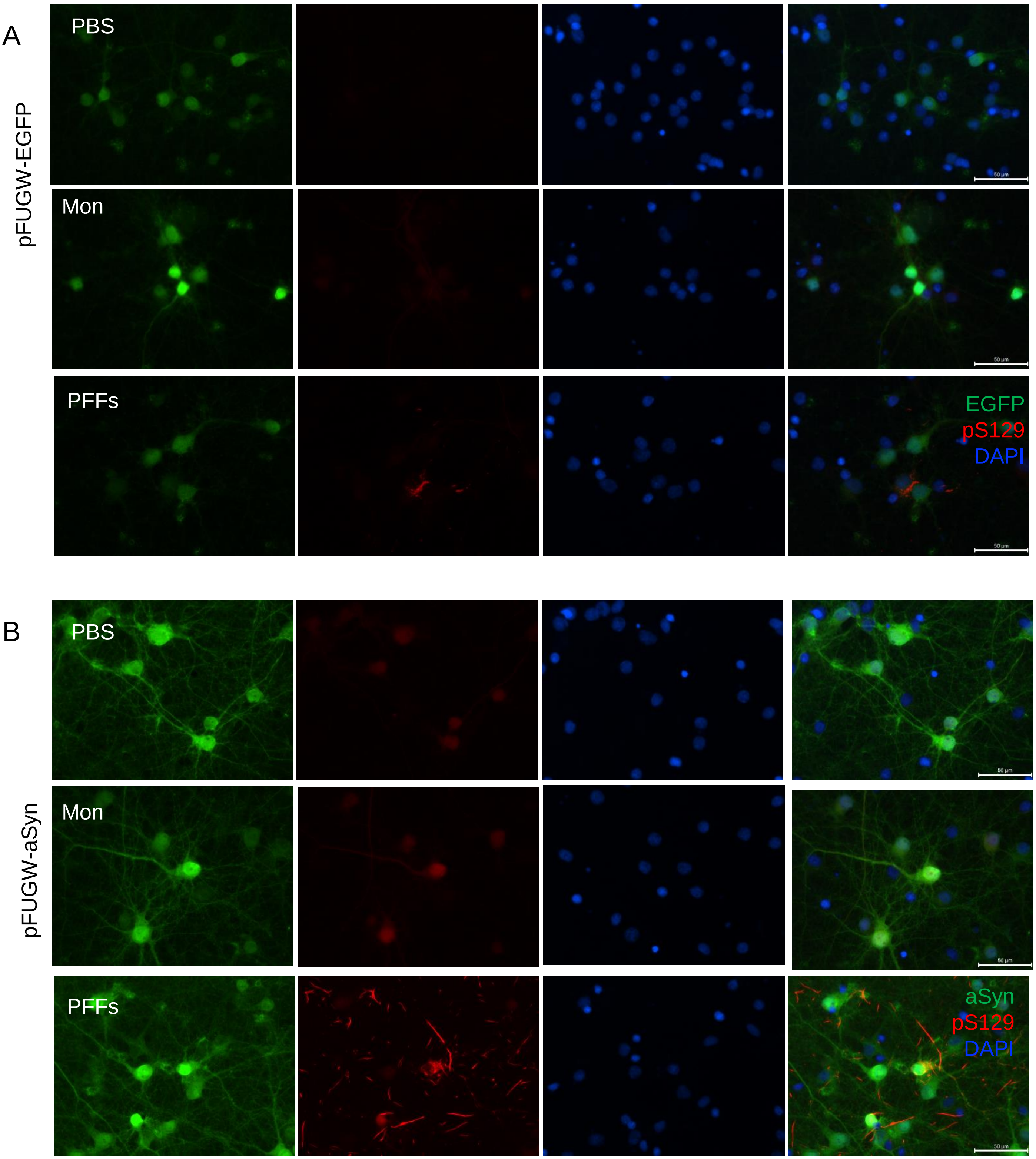

Supplementary Figure 7

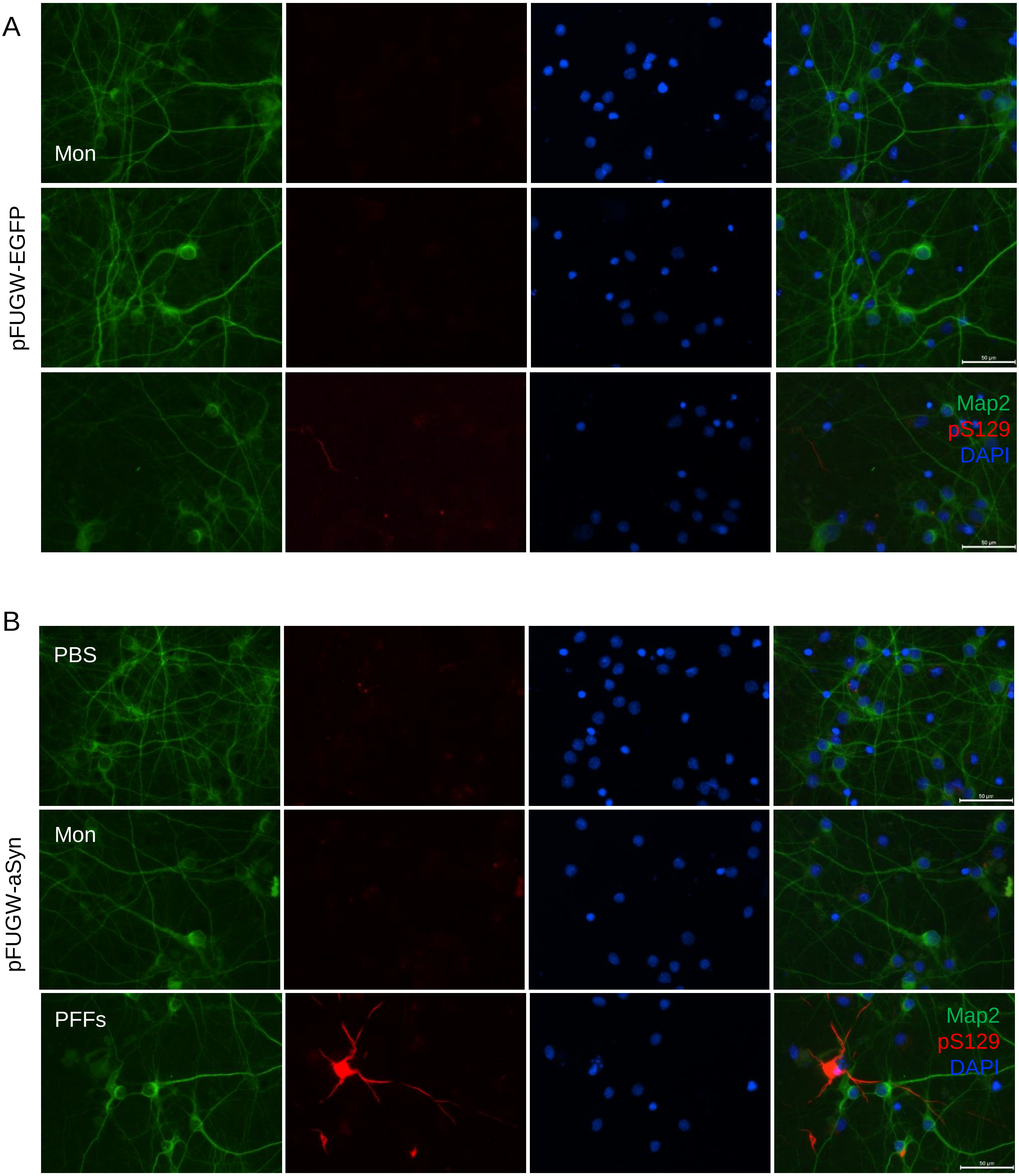

Supplement: Supplementary file 1 — Supplementary file1 Supplementary Figure 1. Exogenous aSyn PFFs induce aSyn aggregation. Comparison between different PFFs. A. Representative SDS-PAGE image of the recombinant proteins used for further experiments. Western blot analysis of 100ng re-combinant aSyn proteins (monomers and PFFs) loaded in SDS-PAGE Gel 12%. B. Electron micrographs of PFFs before (A) and after (B) sonication (Scale bars 500 nm) [6]. C. Treat-ment with recombinant aSyn from different origin and quantification of the percentage of cells with aSyn inclusions. For these experiments we treated the cell with recombinant aSyn produced in the laboratory of Dr. Melki (PFFs 1= our own material and PFFs 2 = Melki’s material; n=3, mean ± SD). Production of human recombinant monomeric WT aSyn and fibrils assembly was prepared as previously described [33]. Supplementary Figure 2. RT-QuIC control experiments using different amounts of PFFs. A. RT-QuIC amplification of aSyn in the presence of different cell lines non-treated with aSyn monomers and PFFs (n=4). Normalized ThT fluorescence intensity is generally lower than that for cells treated with monomers and PFFs. B. Effect of different PFFs quantities in the RT-QuiC amplification of 1 µg of monomeric aSyn. Higher PFFs quantity leads to shorter lag times and higher overall normalized ThT fluorescence levels (n=3). C. Initial normalized ThT fluorescence intensity values among cell lines shows no significant differences between the different groups (n=4). D. RT-QuIC from the different cell lines treated with aSyn monomers and PFFs, showing all the additional controls for the reaction (n=4). Supplementary Figure 3. Representative images from fixed HEK293-aSynEGFP (A) and HEK293-aSynBiFC (B) cells after treatment with PBS, aSyn monomers and PFFs. Immunostaining with an anti-p-Ser129 (red) antibody. DAPI was used for nuclear staining (Scale bar 25 µm). Supplementary Figure 4. Representative immunoblot showing the expression levels of aSyn from the HEK293-aS [file 12035_2021_2713_MOESM1_ESM.pdf]
